# Determination of Vitamin C (Ascorbic Acid) Using High Performance Liquid Chromatography Coupled with Electrochemical Detection

**DOI:** 10.3390/s8117097

**Published:** 2008-11-07

**Authors:** Zbynek Gazdik, Ondrej Zitka, Jitka Petrlova, Vojtech Adam, Josef Zehnalek, Ales Horna, Vojtech Reznicek, Miroslava Beklova, Rene Kizek

**Affiliations:** 1 Department of Breeding and Propagation of Horticultural Plants, Faculty of Horticulture, Valticka 337, CZ-691 44 Lednice, Faculty of Agronomy, Zemedelska 1, CZ-613 00 Brno, Mendel University of Agriculture and Forestry, Czech Republic; 2 Department of Agrochemistry, Soil Science, Microbiology and Plant Nutrition, Faculty of Agronomy, Zemedelska 1, CZ-613 00 Brno, Mendel University of Agriculture and Forestry, Czech Republic; 3 Department of Chemistry and Biochemistry, Faculty of Agronomy, Zemedelska 1, CZ-613 00 Brno, Mendel University of Agriculture and Forestry, Czech Republic; 4 Department of Animal Nutrition and Forage Production, Faculty of Agronomy, Zemedelska 1, CZ-613 00 Brno, Mendel University of Agriculture and Forestry, Czech Republic; 5 Tomas Bata University, T.G. Masaryka 275, CZ-762 72 Zlin, Czech Republic; 6 Department of Veterinary Ecology and Environmental Protection, Faculty of Veterinary Hygiene and Ecology, University of Veterinary and Pharmaceutical Sciences, Palackeho 1-3, CZ-612 42 Brno, Czech Republic

**Keywords:** Ascorbic Acid, Flow Injection Analysis, High Performance Liquid Chromatography, Electrochemical Detection, Fruits, Pharmaceutical Preparation, Human Blood Serum

## Abstract

Vitamin C (ascorbic acid, ascorbate, AA) is a water soluble organic compound that participates in many biological processes. The main aim of this paper was to utilize two electrochemical detectors (amperometric – Coulouchem III and coulometric – CoulArray) coupled with flow injection analysis for the detection of ascorbic acid. Primarily, we optimized the experimental conditions. The optimized conditions were as follows: detector potential 100 mV, temperature 25 °C, mobile phase 0.09% TFA:ACN, 3:97 (*v*/*v*) and flow rate 0.13 mL·min^-1^. The tangents of the calibration curves were 0.3788 for the coulometric method and 0.0136 for the amperometric one. The tangent of the calibration curve measured by the coulometric detector was almost 30 times higher than the tangent measured by the amperometric detector. Consequently, we coupled a CoulArray electrochemical detector with high performance liquid chromatography and estimated the detection limit for AA as 90 nM (450 fmol per 5 μL injection). The method was used for the determination of vitamin C in a pharmaceutical preparations (98 ± 2 mg per tablet), in oranges (*Citrus aurantium*) (varied from 30 to 56 mg/100 g fresh weight), in apples (*Malus sp.*) (varied from 11 to 19 mg/100 g fresh weight), and in human blood serum (varied from 38 to 78 μM). The recoveries were also determined.

## 1. Introduction

### 1.1 Biological function of vitamin C

**Figure 1.**
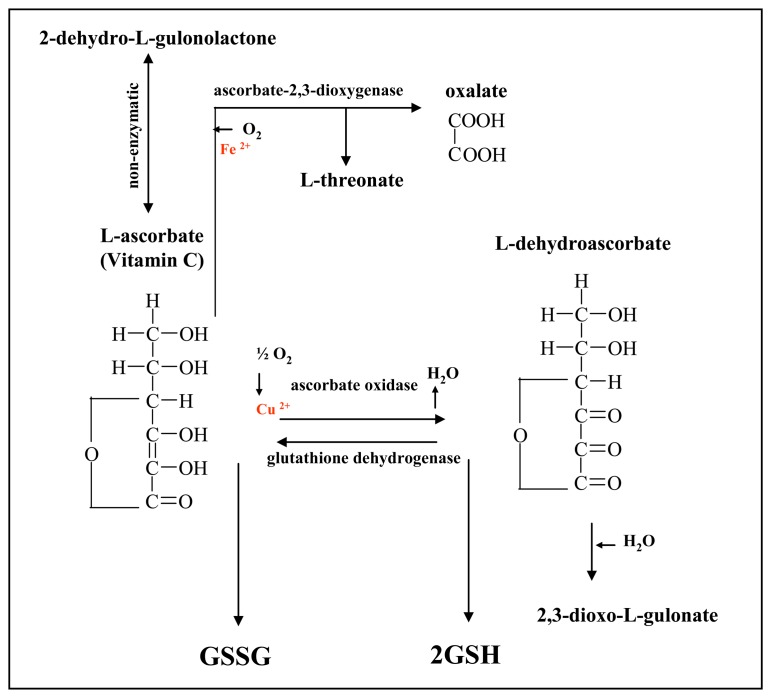
Scheme of a biological function of ascorbic acid (GSH – reduced glutathione, GSSG – oxidized glutathione).

Vitamin C (ascorbic acid, ascorbate, AA) is a water soluble organic compound involved in many biological processes (Figure 1). AA plays crucial roles in electron transport, hydroxylation reactions and oxidative catabolism of aromatic compounds in animal metabolism [1]. Although all the functions of AA are not fully explained, it is likely that it is also involved in maintaining the reduced state of metal cofactors, for example at monooxygenase (Cu^+^) and dioxygenase (Fe^2+^) [2]. In cells the other role of AA is to reduce hydrogen peroxide (H_2_O_2_), which preserves cells against reactive oxygen species [3-5]. An oxidation cycle of ascorbic acid to dehydroascorbic acid is shown in Figure 1. The details about ascorbic acid antioxidant system cooperated with glutathione was described by Meister [6]. Besides this, primates and several other mammals are not able to synthesise ascorbic acid [5]. The animal species, which are able to produce this molecule, biosynthesise AA from glucose catalyzed Lgulonolactonoxidase [1,2]. In spite of the ability to synthesize this molecule both groups of animal species suffer from deficiency of AA [1,2].

### 1.2 Daily needs of vitamin C

The only way humans uptake ascorbic acid is via food [7], but the daily needs of vitamin C for a human are not clear yet. Linus Pauling postulated that people's needs for vitamins and other nutrients vary markedly and that to maintain good health, many people need amounts of nutrients much greater than the recommended doses. According to his suggestions, daily uptake of vitamin C has to be within units of grams of AA to reduce the incidence of colds and other diseases. These “huge” amounts of AA have not been ever proved as the reason for large reducing of the incidence of illnesses. Nowadays the estimated average requirement and recommended dietary allowance of ascorbic acid are 100 mg per day and 120 mg per day, respectively [8,9].

### 1.3 Content of vitamin C in foods

AA can be mostly found in fruits and vegetables. The main sources of AA are citrus fruits, hips, strawberries, peppers, tomatoes, cabbage, spinach and others [3]. If one wants to uptake AA from animal sources, liver and kidney are the tissues with highest contents of this molecule, but in comparison with plant sources the amount of AA is very low [10]. The content of AA in food can be affected by many factors such as clime, method of harvest, storing and processing. Thus, there is a need of analytical procedures able to not only monitor AA content in agricultural and food products, but also in body liquids and tissues [11]. Authors also paid their attention at detection of AA in blood serum [12-15].

### 1.4 Methods for ascorbic acid determination

Many analytical techniques including sensors and biosensors [16-18] have been suggested for a detection of ascorbic acid in very varied types of samples. Hyphenated instruments consisting of flow injection analysis [19-22], high performance liquid chromatography [23-25] or capillary electrophoresis [26-29] instruments and a detector are mostly utilized for the determination of AA. However, some of these methods are time-consuming, some are costly, some need special training operators, or they suffer from the insufficient sensitivity or selectivity. Limits of detection ranged from μM [30-32] to nM [33-36] and lower [12].

Electrochemical detection is an attractive alternative method for detection of electroactive species, because of its inherent advantages of simplicity, ease of miniaturization, high sensitivity and relatively low cost. Electrochemical detection typically worked in amperometric or coulometric mode can be coupled with liquid chromatography to provide high sensitivity to electroactive species. The main aim of this paper is to utilize two electrochemical detectors (amperometric – Coulouchem III and coulometric – CoulArray) coupled with flow injection analysis for detection of ascorbic acid. The more sensitive technique is further applied on analysis of real samples (pharmaceutical preparation, oranges and apples fruits, and human blood serum).

## 2. Material and Methods

### 2.1 Chemicals, material and pH measurements

HPLC-grade acetonitrile (>99.9%; v/v) from Merck (Darmstadt, Germany) was used. Other chemicals used were purchased from Sigma-Aldrich (St. Louis, USA) in ACS purity unless noted otherwise. Stock standard solutions of the AA (100 mM) were prepared with ACS water (Sigma-Aldrich, USA) and stored in the dark at -20 °C. Working standard solutions were prepared daily by dilution of the stock solutions. The stability of AA in samples is strongly influenced by oxygen, which oxidises AA to dehydroascorbic acid. To avoid direct an oxidation reducing agents or acidification by acids can be used [37]. Here, we used dithiothreitol (DTT). All solutions were filtered through 0.45 μm Nylon filter discs (Millipore, Billerica, Mass., USA) prior to HPLC analysis. The pH value was measured using WTW inoLab (Weilheim, Germany), controlled by software MultiLab Pilot. The pHelectrode was regularly calibrated with WTW buffers (Weilheim, Germany).

### 2.2 Flow injection analysis/High performance liquid chromatography with CoulArray or Coulochem electrochemical detector

#### CoulArray

The FIA/HPLC-ED system consisted of two solvent delivery pumps operating in the range 0.001-9.999 mL·min^-1^ (Model 582 ESA Inc., Chelmsford, MA), a reaction coil (1 m) and/or Metachem Polaris C18A reversed-phase column (150.0 × 2.1 mm, 3 μm particle size; Varian Inc., CA, USA) and a CoulArray electrochemical detector (Model 5600A, ESA, USA). The electrochemical detector includes two flow cells (Model 6210, ESA, USA). Each cell consists of four analytical cells containing working carbon porous electrode, two auxiliary and two reference electrodes. Working electrodes were polished electrochemically applying of positive/negative potential cycles (1/-1 V) at increased flow of the mobile phase (1 mL·min^-1^). Both the detector and the reaction coil/column were thermostated. The sample (5 μL) was injected manually.

#### Coulochem III

The FIA-ED system consisted of a solvent delivery pump operating in range of 0.001-9.999 mL·min^-1^ (Model 583 ESA Inc., Chelmsford, MA, USA), a guard cell (Model 5020 ESA, USA), a reaction coil (1 m) and an electrochemical detector. The electrochemical detector (ED) includes one low volume flow-through analytical cells (Model 5040, ESA, USA), which is consisted of glassy carbon working electrode, palladium electrode as reference electrode and auxiliary carbon electrode, and Coulochem III as a control module. A glassy carbon electrode was polished mechanically by 0.1 μm of alumina (ESA Inc., USA) and sonicated at the laboratory temperature for 5 min using a Sonorex Digital 10 P Sonicator (Bandelin, Berlin, Germany) at 40 W as it was described by [38]. The sample (5 μL) was injected manually. The obtained data were treated by CSW 32 software. The experiments were carried out at room temperature.

### 2.3 Preparation of real samples

*The pharmaceutical preparation (a tablet)* – Celaskon (Leciva, Prague, Czech Republic) was ground in a mortar (*n* = *5*). Then, ground powder (about 1 mg) was dissolved in ACS water (1 mL). Oranges and apples (*Citrus aurantium* and *Malus sp.*) were bought at TESCO stores (*n* = *5*). The pericarps of the fruits were removed, and then the fruits (app. 0.25 g) were homogenized using a mortar. The extracts obtained were filtered through filter paper (Niederschlag, Germany), transferred into a volumetric flask and diluted with ACS water. Measurements of the samples were carried out immediately after preparation steps. *Human blood serum samples* were obtained from the Department of Clinical Biochemistry, Trauma Hospital Brno (Czech Republic), (*n* = *10*). Human sera were frozen at –20 °C immediately after collection. The samples were 100 × diluted with ACS water and filtered through 0.45 μm Teflon membrane filter prior to measurement.

### 2.4 Accuracy, precision and recovery

Accuracy, precision and recovery of AA were evaluated with homogenates (human blood serum, a fruit and Celaskon tablets) spiked with the standard. Before extraction, AA standards (100 μL) and water (100 μL) were added to the homogenates of real samples. The homogenates were assayed blindly and AA concentrations were derived from the calibration curves. Accuracy was evaluated by comparing estimated concentrations with known concentrations of AA. Calculation of accuracy (% Bias), precision (% C.V.) and recovery was carried out as indicated by [39-41].

### 2.5 Descriptive statistics

Data were processed using MICROSOFT EXCEL® (USA). Results are expressed as mean ± S.D. unless noted otherwise. The detection limits (3 signal/noise, S/N) were calculated according to Long and Winefordner [42], whereas N was expressed as standard deviation of noise determined in the signal domain unless stated otherwise.

## 3. Results and Discussion

Stationary and flow electrochemical techniques are very attractive instruments for determining various biologically important compounds such as proteins [43-60], organic compounds of plant origin [61-66], drugs [67-70], etc. Here, we aimed at utilizing two different electrochemical detectors – amperometric (Coulouchem III) or coulometric (CoulArray) coupled with high performance liquid chromatography for detection of ascorbic acid.

### 3.1 Flow injection analysis coupled with CoulArray electrochemical detector to detect ascorbic acid

An electrochemical behaviour of AA at the surface of working electrodes was investigated. FIA enables us to optimise experimental conditions for analytical determination of AA easily and rapidly. Primarily, the influence of potential applied to single working electrodes on oxidation signal of AA was studied. The potential varied from 100 to 400 mV and signal of various concentration of AA (12.5, 25, 50, 100, 200, 300, 400, 500 and 1000 μM) was measured.

**Figure 2.**
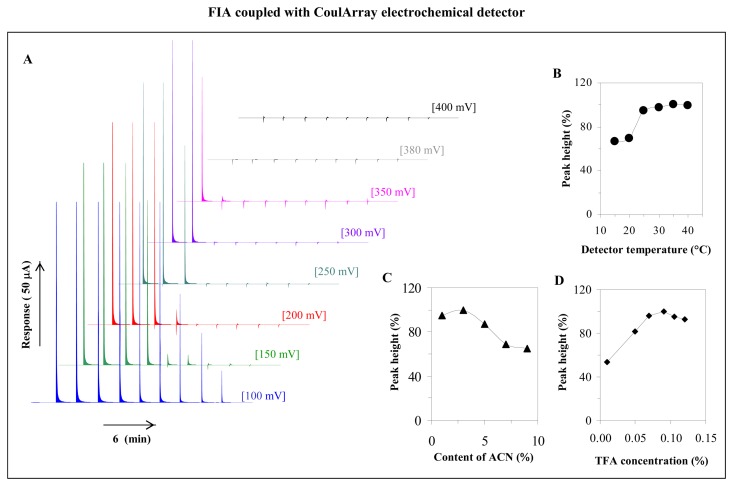
FIA coupled with CoulArray electrochemical detector. FIA-ED full scan of ascorbic acid (12.5, 25, 50, 100, 200, 300, 400, 500 and 1000 μM). Detector electrodes potentials at full scan: 100, 150, 200, 250, 300, 350, 380 and 400 mV (**A**). Dependence of ascorbic acid peak height on detector temperature (**B**), on content of acetonitrile in mobile phase consisted from acetonitrile and trifluoroacetic acid (**C**) and on TFA concentration (**D**). FIA-ED parameters: flow rate of mobile phase – 0.1 mL·min^-1^; ascorbic acid concentrations – 100 μM; 5 μL samples was injected.

Each concentration belongs to the signal shown in FIA-ED record in Figure 2A. Apparently maximal current responses were measured at the surface of the first working electrode. This phenomenon was observed at all concentrations of AA measured by FIA-ED. Moreover it can be concluded that lower concentration of AA was measured, the signal disappeared earlier (Figure 2A). This phenomenon associates with i) type of detector used because CoulArray detector operates in coulometry mode and coulometric detector promotes near 100 % electrochemical conversion of analytes below the designated potential of the detector, and with ii) easy electrochemical oxidation of AA at the surface of the working electrodes already under low potentials (about 100-200 mV). For choosing of the optimal potential for detection of AA, we applied the potential scale from 100 to 400 mV per 50 mV at all eight electrodes and measured the signal at the first electrode only. Based on these results, the most suitable potential for detection of AA was 100 mV.

Further, the affecting height of the signal of AA by temperature was studied. The temperature within the range from 15 to 40 °C was tested. The dependence obtained is shown in Figure 2B. The peak height increased for more than 30 % at 25 °C compared to signals obtained at lower temperatures (15 and 20 °C). At temperatures higher than 25 °C very low changes in the height was observed (Figure 2B). Therefore temperature of 25 °C was used in the following experiments.

It is commonly known that electrochemical analysis needs the presence of an electrolyte, although the presence of a non-aqueous solvent in a mobile phase is needed for successful and rapid simultaneous determination of compounds of interest. These non-aqueous solvent negatively influence electrochemical analysis [41,71]. Therefore, affecting of AA signal by the ratio of acetonitrile:trifluoroacetic acid (ACN:TFA, *v*/*v*) was investigated (Figure 2C). We did not determine any changes in repeatability with increasing content of ACN in mobile phase. The highest signal was measured at 3% (*v*/*v*) content of ACN in the mobile phase.

It is a common knowledge that trifluoroacetic acid (TFA) is ion pairing agent in liquid chromatography that can react with the vitamin and may assign them charge. Thus, we selected a mixture of acetonitrile with aqueous solution of trifluoroacetic acid at ratio 3:97 (*v*/*v*) and tested the influence of various concentrations of TFA. We found out that the response increased to 0.005% concentration of TFA, then slightly decreased. The highest signal of AA was measured at 0.09% TFA:ACN, 3:97 (*v*/*v*) (Figure 2D). Moreover, flow rate of the mobile phase is other important parameter influencing peak height. We tested the flow of mobile phase from 0.05 to 0.2 mL·min^-1^. The highest current responses were obtained at flow 0.13 mL·min^-1^.

**Figure 3.**
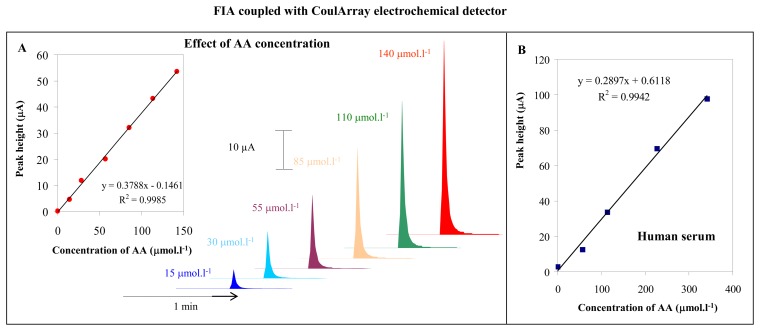
Effect of AA concentration. Dependence of peak height on different AA concentrations (15, 30, 55, 85, 110 and 140 μM) (**A**). Human blood serum. Influence of biological matrix on AA signal. Concentration of AA – 57, 114, 227 and 341 μM (**B**). FIA-ED parameters were as follows: mobile phase – acetonitrile and 0.09% trifluoroacetic acid in ratio 3:97; detector temperature – 25 °C; flow rate of mobile phase – 0.13 mL·min^-1^; 5 μL samples was injected.

The optimal experimental conditions are: detector potential 100 mV, temperature 25 °C, mobile phase 0.09% TFA:ACN, 3:97 (*v*/*v*) and its flow rate 0.13 mL·min^-1^. Under these conditions we measured the dependence of AA peak height on its concentration. The peaks obtained were well developed and symmetrical (insets in Figure 3A). The calibration curve is shown in Figure 3A (y = 0.3788x – 0.1461; R^2^ = 0.9985). The detection limit (3 S/N) for AA was evaluated as 100 fmol per 5 μL injection. Further the method was utilized for determination of AA spiked into human blood serum samples. AA was spiked into blood serum (100 × diluted) and electrochemical responses were observed. The obtained calibration curve (y = 0.2897x + 0.6118; R^2^ = 0.9942) is shown in Figure 3B. The detection limit (3 S/N) was evaluated as 20 pmol for a 5 μL injection.

### 3.2 Flow injection analysis coupled with Coulochem III electrochemical detector to detect AA

**Figure 4.**
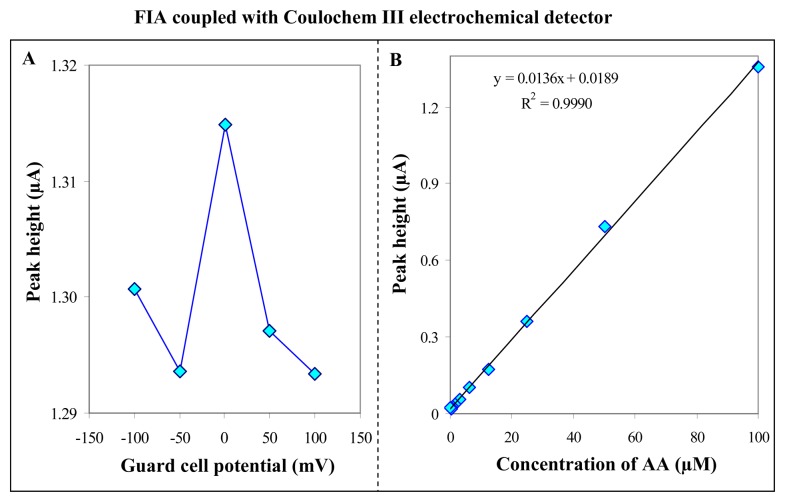
FIA coupled with Coulochem III electrochemical detector. Dependence of peak height on Guard cell potentials (-100, -50, 0, 50 and 100 mV) (**A**) and different AA concentrations (15, 30, 55, 85, 110 and 140 μmol·L^-1^) (**B**). FIA-ED parameters were the same as shown in Figure 3.

Coulochem III is an amperometric detectors, which are somewhat inefficient because only a fraction of analyte (typically 5–10 %), which passes over the working electrode, actually diffuses onto the electrode surface and experiences electrochemical conversion [72]. One may suggest that coulometric detector CoulArray should be 10–20 times more sensitive than amperometric detection simply because of improved mass transfer. Unfortunately the increased conversion efficiency of the analyte is accompanied by a similar increase for the electrolyte (background) reactions, and nearly no lowering of detection limits is realized [72]. Moreover, amperometry can provide better LOD in terms of concentrations with miniaturized cells. Therefore we adopted above optimized experimental conditions to detect AA by FIA coupled Coulochem III electrochemical detector and aimed on comparison of amperomeric and coulometric detectors. Unlike CoulArray amperometric detector Coulochem contains a device called “Guard cell”, which can oxidize electroactive impurities in mobile phase and, thus, lower the noise. We tested five potentials (-100, -50, 0, 50 and 100 mV) applied on Guard cell and measured the signal of AA. The dependence obtained is shown in Figure 4A. Based on the results obtained the optimal Guard cell potential was 0 mV. The potentials lower or higher than the optimal value slightly decreased the signal of AA. This phenomenon can be associated to easy electrochemical conversion of the target molecule already at Guard cell. To compare the detectors we again measured the dependence of AA concentration on peak height (Figure 4B). The equation of the calibration curve obtained was y = 0.0136x + 0.0189, with R^2^ = 0.9990.

The concentration range of AA analysed using both coulometric and amperometric detectors was almost the same due to possibility of comparing of the sensitivity of the instruments. The tangents of the calibration curves were 0.3788 for coulometric and 0.0136 for amperometric. Based on these results the tangent of calibration curve for AA measured using coulometric detector was almost 30 times higher than the tangent measured by amperometric detector. Coulometric detector is much more sensitive to presence of AA, thus, we utilized this detector in following experiments.

### 3.3 High performance liquid chromatography coupled with CoulArray electrochemical detector to detect AA

**Figure 5.**
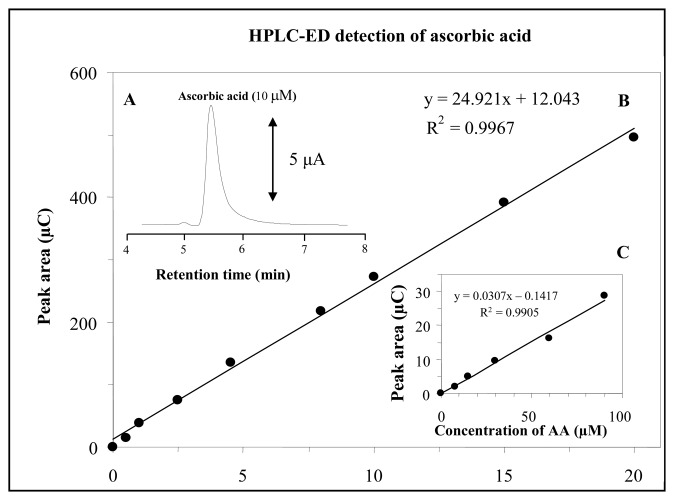
HPLC-ED detection of ascorbic acid HPLC-ED chromatogram of ascorbic acid (10 μM) (**A**). Dependence of peak area on different AA concentrations in the range from 0.5 – 20 mM (**B**) and 10 – 90 μM (**C**). HPLC-ED parameters were as follows: chromatographic column – MetaChem Polaris C18A (150 × 2.0 mm, 3 μm particle size) mobile phase – acetonitrile and 0.09% trifluoroacetic acid in ratio 3:97; detector temperature – 25 °C; flow rate of mobile phase – 0.13 mL·min^-1^; all detectors potential – 150 mV; 5 μL samples was injected.

Under the optimized conditions mentioned above ascorbic acid was measured using high performance liquid chromatography coupled with CoulArray electrochemical detector (HPLC-ED). The retention time of AA was 5.4 min. (Figure 5A). Dependence of peak area on AA concentration was strictly linear and relative standard deviation (R.S.D.) was about 6 % (*n* = *5*). Equation of calibration curve measured within the range from 0.5 to 20 mM of AA was y = 24.921x + 12.043 with R^2^ = 0.9967 (Figure 5B). In addition we attempted to analyse lower concentration range from 10 to 90 μM. The calibration curve obtained was y = 0.0307x – 0.1417; R^2^ = 0.9905 (Figure 5C). Using the optimized HPLC-ED it was possible to detect nanomolar concentrations of AA (LOD: 90 nM; 450 fmol per 5 μL injection).

### 3.4 Analysis of real samples

**Figure 6.**
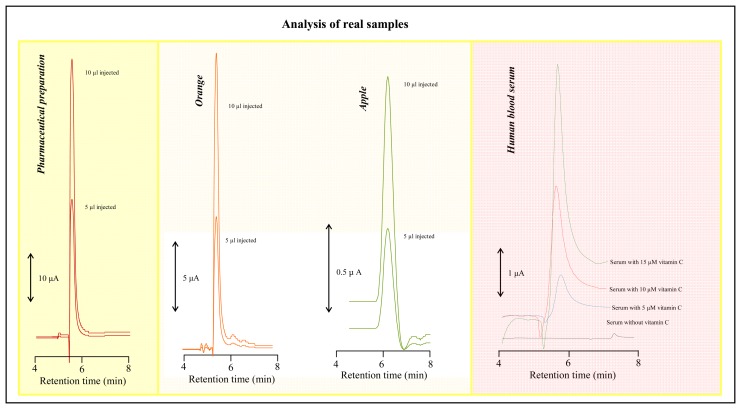
Typical HPLC-ED chromatograms of pharmaceutical preparation, extracts from apples and oranges, and human blood serum. For HPLC-ED parameters see caption of Figure 5.

The concentration of ascorbic acid was determined in pharmaceutical preparation, two species of fruits and human blood serum under the optimized experimental conditions using HPLC coupled with CoulArray electrochemical detector. Typical HPLC-ED chromatograms of the fore-mentioned samples are shown in Figure 6. We determined the concentration of ascorbic acid as 98 ± 2 mg per one tablet in a pharmaceutical preparation called Celaskon. The manufacturer of this preparation declares the amount of AA as 100 mg per tablet. The recovery of the amount of AA added into the sample was 105 % for lower addition of AA (5 μg·mL^-1^) and 95 % for higher addition of AA (15 μg·mL^-1^); for more details see in Table 1. Moreover we used HPLC-ED to determine AA concentration in fruits species. We found that AA amount in oranges (*Citrus aurantium*) varied in the range from 30 to 56 mg/100 g of fresh weight and in apples (*Malus sp.*) from 11 to 19 mg/100 g of fresh weight. The recovery of AA measured in the homogenate prepared from fruits *Citrus aurantium* was 103 % for lower addition (5 μg·mL^-1^) and 104 % for higher addition (15 μg·mL^-1^); Table 1. To evaluate HPLC-ED technique for analysis of human body liquids we spiked human blood serum and found out that recovery of AA varied from 102 to 98 % according to lower (5 μg·mL^-1^) or higher (15 μg·mL^-1^) content of AA. The tested blood sera contained AA within the range from 38 to 78 μM.

## 4. Conclusions

High performance liquid chromatography coupled with an eight channel electrochemical detector appears to be a very suitable analytical instrument for sensitive ascorbic acid determination. Using the optimized technique ascorbic acid was determined in pharmaceutical preparations, fruits and human blood serum samples.

## Figures and Tables

**Table 1.** Recovery of AA for orange fruit (*Citrus aurantium*), Celaskon tablet and human serum sample analysis (*n* = 3).

**Sample**	**Homogenate(μg mL1)**^a^, ^b^, ^c^	**Spiking AA(μg mL^1^)**^a^, ^b^	**Homogenate+spikingAA (μg mL^-1^)**^a^, ^b^	**Recovery(%)**
*Celaskon (tablets)*	9.8 ± 0.2 (2.0)	5.0 ± 0.2 (4.0)	15.6 ± 0.9 (5.8)	105
		15.9 ± 0.9 (5.7)	24.3 ± 1.9 (7.8)	95

*Citrus aurantium*	4.3 ± 0.1 (2.3)	5.1 ± 0.1 (1.9)	9.7 ± 0.3 (3.1)	103
		15.3 ± 1.0 (6.5)	20.3 ± 1.6 (7.9)	104

*Human serum*	7.1 ± 0.3 (4.2)	4.8 ± 0.2 (4.2)	12.1 ± 0.6 (5.0)	102
		15.1 ± 0.9 (6.0)	21.7 ± 1.8 (8.3)	98

a amount of AA

b the results are expressed as mean ± S.D. (C.V. %)

c re-computation of AA molar concentration on weight concentration – 57 μM is 10 μg·mL^-1^
